# Generation of *Epichloë* Strains Expressing Fluorescent Proteins Suitable for Studying Host-Endophyte Interactions and Characterisation of a T-DNA Integration Event

**DOI:** 10.3390/microorganisms8010054

**Published:** 2019-12-27

**Authors:** Inoka K. Hettiarachchige, Emma J. Ludlow, Piyumi N. Ekanayake, Natasha D. Brohier, Sareena Sahab, Timothy I. Sawbridge, German C. Spangenberg, Kathryn M. Guthridge

**Affiliations:** 1 Agriculture Victoria, AgriBio, Centre for AgriBioscience, Bundoora, VIC 3083, Australia; inoka.hettiarachchige@agriculture.vic.gov.au (I.K.H.); emma.ludlow@agriculture.vic.gov.au (E.J.L.); piyumi.ekanayake@agriculture.vic.gov.au (P.N.E.); natasha.brohier@agriculture.vic.gov.au (N.D.B.); sareena.sahab@agriculture.vic.gov.au (S.S.); tim.sawbridge@agriculture.vic.gov.au (T.I.S.); german.spangenberg@agriculture.vic.gov.au (G.C.S.); 2 School of Applied Systems Biology, La Trobe University, Bundoora, VIC 3083, Australia

**Keywords:** *Epichloë*, endophyte, reporter gene, green fluorescent protein, DsRed, *A. tumefaciens*-mediated transformation, transformants, T-DNA integration, sequencing

## Abstract

Methods for the identification and localisation of endophytic fungi are required to study the establishment, development, and progression of host-symbiont interactions, as visible reactions or disease symptoms are generally absent from host plants. Fluorescent proteins have proved valuable as reporter gene products, allowing non-invasive detection in living cells. This study reports the introduction of genes for two fluorescent proteins, green fluorescent protein (GFP) and red fluorescent protein, DsRed, into the genomes of two distinct perennial ryegrass (*Lolium perenne* L.)-associated *Epichloë* endophyte strains using *A. tumefaciens*-mediated transformation. Comprehensive characterisation of reporter gene-containing endophyte strains was performed using molecular genetic, phenotypic, and bioinformatic tools. A combination of long read and short read sequencing of a selected transformant identified a single complex T-DNA insert of 35,530 bp containing multiple T-DNAs linked together. This approach allowed for comprehensive characterisation of T-DNA integration to single-base resolution, while revealing the unanticipated nature of T-DNA integration in the transformant analysed. These reporter gene endophyte strains were able to establish and maintain stable symbiotum with the host. In addition, the same endophyte strain labelled with two different fluorescent proteins were able to cohabit the same plant. This knowledge can be used to provide the basis to develop strategies to gain new insights into the host-endophyte interaction through independent and simultaneous monitoring in planta throughout its life cycle in greater detail.

## 1. Introduction

Asexual endophytes from the genus *Epichloë* (previously *Neotyphodium)*, are strictly seed-transmitted endophytic symbionts of cool-season grasses (Poaceae, sub-family Pooideae) characterised by a life-cycle wholly confined to the host plant [1,2]. The relationships between asexual *Epichloë* species and their grass hosts are often mutualistic in nature [3]. Endophyte infection can impart environmental stress tolerance and protection from herbivory, thus markedly enhancing host survival. The latter property is attributable to production of bioprotective alkaloid compounds, including ergot alkaloids, pyrrolopyrazines (such as peramine), aminopyrrolizidines (such as lolines), and indole-diterpenes (including lolitrems and epoxy-janthitrems) [3,4,5,6]. Conversely, the endophyte obtains nutrition and shelter from the host [3]. Several taxa of asexual *Epichloë* fungal endophytes have been found to form associations with perennial ryegrass, the most important pasture grass species for temperate grassland agriculture, including *E. festucae* var. *lolii* (*Lp*TG-1, *Lolium perenne* taxonomic group 1), *Lp*TG-2, *Lp*TG-3, and *Lp*TG-4, respectively [7,8].

A considerable number of studies have been conducted describing the production of alkaloids by endophyte-forage grass associations, primarily due to the economic impact of deleterious metabolites on agricultural systems [9]. However, much less is known about other aspects of the association, such as host colonisation, cross species compatibility, capacity for co-existence of multiple endophytes of the same and different species within the same host, and subsequent vegetative and intergenerational stability. Methods for identification and localisation of endophytes are required in order to study the establishment, development, and progression of host-symbiont interactions, as visible reactions or disease symptoms are generally absent from host plants [10]. Fluorescent proteins such as GFP and DsRed are extremely useful markers in living cells and organisms [11]. Fungal strains that express these fluorescent proteins provide the ability to directly visualise biological processes, such as host colonisation and establishment of associations, without obvious effects on fungal growth [12]. Complementary use of these reporter proteins is highly advantageous, as conventional fluorescence microscopy can effectively distinguish between co-expressed DsRed and GFP, due to considerable differences in spectral properties, in contrast to different GFP variants in multicolour experiments [13]. Several studies of genetically modified *Epichloë* endophytes expressing GFP have been reported [14,15,16]. However, reporter gene-containing endophytes of different taxa expressing both GFP and DsRed and capable of colonising perennial ryegrass have not been described. Strains of this kind would be highly valuable for analysis of processes involved in various aspects of the host–endophyte association, such as time-course analysis of endophyte host colonisation, interactions between co-inoculated endophytes, and the possibility of observing in planta parasexual events based on inter-strain hyphal fusion.

Molecular characterisation of fungi that express fluorescent proteins have been the subject of extensive study [17,18,19]. PCR-based techniques and Southern hybridisation analysis have been used for identification of transgene-flanking sequences in several fungal species [20,21,22]. However, these techniques are of limited use for identification of multiple complex patterns of T-DNA integrations, or incomplete sequences [23,24,25]. Therefore, until recently, limited information was available on the patterns and sites of T-DNA integration in fungi following *A. tumefaciens*-mediated transformation, although some characterisation of T-DNA insertion events based on PCR and Southern hybridisation has been reported for *Saccharomyces cerevisiae*, *Leptosphaeria maculans, Magnaporthe oryzae*, *Histoplasma capsulatum, Fusarium oxysporum,* and *Cryptococcus neoformans* var. *neoformans* [20,21,26,27,28].

High-throughput second-generation sequencing can be used to effectively and accurately determine exact transgene copy number, presence or absence of the vector backbone, as well as the location of specific transgene integration sites [29,30]. However, generation of accurate genome assemblies, with large repeat structures, using short read sequencing is challenging [31]. In contrast, long read sequencing such as the Oxford Nanopore Technologies MinION have the ability to overcome these limitations and have been used to investigate complex T-DNA integration patterns in plants recently [31]. Undesired, complex, and unanticipated T-DNA integrations have been frequently observed for transgenic plants; precise and detailed analysis of the genome following any form of genetic modification is critical for commercial, regulatory, and research purposes [31,32]. Comprehensive characterisation of changes in genome configuration following T-DNA integration is also important as this may also impact expression of the transgene [33]. The importance has become further emphasized considering the use of *A. tumefaciens*-mediated transformation methods for genome editing experiments [31]. Use of long read sequencing to characterise T-DNA integration events in fungi, including endophytes, has not been reported and hence, represents a novel application of this technology for asexual *Epichloë* fungal endophytes. This study offers new perspectives to understand the complex patterns of T-DNA integration in fungal genomes.

The present study describes the successful generation of GFP and DsRed expressing asexual *Epichloë* endophyte strains, henceforth referred to as endophytes, belonging to different representative taxa, *Lp*TG-3 (strain NEA12) and *Lp*TG-4 (strain E1), which provide bioprotective properties to the host plant against invertebrate herbivores, likely through production of epoxy-janthitrems [6,8,34]. This study also reports comprehensive characterisation of these reporter gene-containing *Epichloë* endophyte strains using molecular genetic, phenotypic, and bioinformatic tools. The ability of these reporter endophytes to establish a successful symbiotum with the host was studied. Furthermore, co-inoculation studies demonstrated the co-existence ability of endophytes expressing GFP and DsRed from the same taxon in the same host. The results obtained in this study strongly suggest the applicability of these reporter gene-containing endophyte strains to better understand host-symbiont association. The outcomes of the first use of third-generation long-range DNA sequencing to identify complex T-DNA integration events in fungal genomes provide a significant advance, which may be further explored to understand the intricacies of *A. tumefaciens*-mediated transformation of fungi.

## 2. Materials and Methods

### 2.1. Microbial Culture Conditions

Properties of the selected endophyte strains are summarised in Table 1. Cultures of NEA12 and E1 were grown either on potato dextrose agar (PDA; Sigma-Aldrich, St. Louis, MO, USA), or in PDB at 22 °C in the dark for a period of 7–10 days, depending upon growth rate. The AGL1 and LBA 4404 strains of *A. tumefaciens* which were used for transformation, and *Escherichia coli* strain DH5α (Thermo Fisher Scientific, Walthman, MA, USA), which was used during construction and maintenance of plasmid constructs, were grown at 28 and 37 °C, respectively, on either LB (Luria–Bertani) agar (5 g yeast extract, 10 g tryptone, 10 g NaCl in 1L of ddH_2_O) plates or in LB broth. PDA and LB media were supplemented with appropriate antibiotics as necessary [35].

### 2.2. Construction of Vectors Containing Reporter Genes

Gene cassettes including the constitutive *Aspergillus nidulans* glyceraldehyde-3-phosphate dehydrogenase promoter (*gpd*P) and the *A. nidulans* tryptophan biosynthesis terminator (*trpC*T), for cloning of the first reading frame A (RFA-A) cassette; the hygromycin B resistance gene (*hph*) under the control of the *A. nidulans trpC* promoter (*trpC*P) and terminator; and reporter genes including *DsRed* (DsRed-Express 736 bp, gb|DQ232603.1|), *sgfp* (gb|EF090408.1|), *egfp* (gb|HQ259114.1|) containing *att*B1 and *att*B2 sites were synthesised and obtained from GeneArt, Regensburg, Germany. The Gateway™-enabled destination vector (pEND0002) was constructed through modifications of the T-DNA region of pPZP200 [36] by cloning the 2.1 kb *Xba*I/*Kpn*I fragment containing the *hph* expression cassette and 2.9 kb *gpd*P-(RFA-A)-*trpC*T cassette (Invitrogen, Thermo Fisher Scientific, Walthman, MA, USA) as previously described in [35].

The 779 bp fragments corresponding to the reporter genes *sgfp* and *egfp* were excised from vectors pMA4 and pMA5, respectively, using the *Sac*I and *Kpn*I restriction enzyme sites. The *DsRed* gene, containing *att*B1 and *att*B2 sites, was excised from the pMA3 vector by co-digestion with the restriction endonucleases *Asc*I and *Pac*I. *DsRed*, *egfp*, and *sgfp* gene fragments containing *att*B1 and *att*B2 sites were cloned into the pDONR221 vector using BP clonase following the manufacturer’s recommended method (Invitrogen, Thermo Fisher Scientific, Walthman, MA, USA) generating entry clones designated pEND0003, pEND0004, and pEND0005, respectively. The entry clones pEND003, pEND004, and pEND005 were combined with the destination vector pEND0002 using a LR clonase reaction following the manufacturer’s recommended method (Invitrogen, Thermo Fisher Scientific, Walthman, MA, USA), to generate the final expression vectors pEND-*DsRed*, pEND-*egfp*, and pEND-*sgfp*, respectively.

T-DNA regions of all expression clones were sequence verified by Sanger sequencing using oligonucleotides; RFA-R1-F (5′-ACAAGGTCGTTGCGTCAGTC-3′), RFA-R1-R (5′-ACATTATACGAGCCGGAAGC-3′), RFA-R2-F (5′-TTATACGCAAGGCGACAAGG-3′), RFA-R2-R (5′-GTAAGCCGGATCCACGCG-3′), PZP-TR-F (5′-CCCACTCCACATCTCCACTC-3′), PZP-TR-R (5′-AAACGCTCTTTTCTCTTAGG-3′), PZP-TL-F (5′-TGTGGTGTAAACAAATTGACG-3′), PZP-TL-R (5′-TTCAATTCGGCGTTAATTCAG-3′), *gpd*P-*trpC*T-F (5′-ATGTCCTCGTTCCTGTCTGC-3′) and *gpdP*-*trpC*T-R (5′-GTCAGCCAACTGCAAACAGA-3′).

Details of all plasmids designed and constructed in this study are summarised in Table S1.

### 2.3. Sensitivity of Non-Transgenic Endophyte Mycelia to Hygromycin B

The sensitivity of non-transformed endophyte mycelia to treatment with hygromycin B were determined by plating 400 µL of liquid culture onto PDA plates containing increasing concentrations of antibiotic (50, 100, 150, 200, 250, and 300 µg/mL) as previously described [35].

### 2.4. Transformation

#### 2.4.1. *A. tumefaciens*-Mediated Transformation

*A. tumefaciens*-mediated transformation of endophyte strains NEA12 and E1 was performed using *A. tumefaciens* cells (AGL1 and LBA 4404) as previously described in [35]. Control experiments were performed by co-cultivation of endophyte mycelia with strains of *A. tumefaciens* that had not been transformed with the plasmid vectors.

#### 2.4.2. Mitotic Stability of Transformed Endophytes

Mitotic stability of putative endophyte transformants obtained from each vector (appearing 2–4 weeks after *A. tumefaciens*-mediated transformation) and protoplast-derived transformants was determined by sub-culturing five successive times, every 2–3 weeks, alternating between media containing and lacking 200 µg/mL hygromycin B as previously described in [35].

#### 2.4.3. Molecular Analysis of Transformed Endophytes

Putative transformants were confirmed by PCR analysis. Genomic DNA was extracted by an optimised procedure described by [37] using 30–60 mg of freeze-dried endophyte mycelium harvested by filtration of liquid culture through Miracloth. PCR analysis was performed using the following primer pairs: GFPF (5′-CCTGAAGTTCATCTGCACCA-3′), GFPR (5′-TCAGGTAGTGGTTGTCG-3′), DsRedF (5′-TCCAAGGTGTACGTGAAGCA-3′), DsRedR (5′-TGGTGTAGTCCTCGTTGTGG-3′), hphF (5′-GATGTTGGCGACCTCGTATT-3′), and hphR (5′- GAATTCAGCGAGAGCCTGAC-3′). PCR cycling conditions consisted of an initial denaturation cycle of 10 min at 95 °C, followed by 30 cycles with 30 s denaturation (95 °C), 30 s annealing (59 °C), and 1 min polymerisation (72 °C).

### 2.5. Preparation and Regeneration of Fungal Protoplasts

Preparation and regeneration of protoplasts was performed as previously described in [6]. In brief, mycelia were harvested and washed 3 times with 30 mL of sterile ddH_2_O and 10 mL of OM buffer (1.2 M MgSO_4_.7H_2_O, 10 mM Na_2_HPO_4_, 100 mM NaH_2_PO_4_.2H_2_O, pH 5.8). Freshly prepared 10 mg/mL Glucanex (30 mL) (Sigma-Aldrich, St. Louis, MO, USA) in OM was added and incubated for 18 h at 30 °C with gentle shaking (80 rpm). Protoplasts were filtered and overlayed with 2 mL of ST buffer (0.6 M sorbitol, 100 mM Tris-HCl, pH 8.0) and centrifuged (Avanti^®^ J-25I; Beckman Coulter, Brea, CA, USA) (5000 rpm for 5 min at 4 °C). Following centrifugation, the protoplasts were removed carefully and STC buffer (1 M sorbitol, 50 mM CaCl_2_·2H_2_O, 50 mM Tris-HCl, pH 8.0) (5 mL) was added. Protoplast pellets were pooled with 5 mL of STC buffer and centrifugation was repeated (5000 rpm for 5 min at 4 °C). The final protoplast pellet was re-suspended in 500 μL of STC buffer.

Serial dilutions of fresh protoplast preparations were made with 40% (*w*/*v*) PEG solution (40% (*w*/*v*) PEG 4000, 1 M sorbitol, 50 mM Tris-HCl, pH 8.0, 50 mM CaCl_2_) in a total volume of 1 mL, followed by gentle mixing and incubation at room temperature for 15 min. Regeneration of protoplasts was performed by spreading molten (50 °C) 0.4% RG II (5 mL) (304 g/L sucrose, 1 g/L KH_2_PO_4_, 1 g/L NH_4_NO_3_, 1 g/L NaCl, 0.25 g/L anhydrous MgSO_4_, 0.13 g/L CaCl_2_.2H_2_O, 1 g/L yeast extract, 12 g/L PD, 1 g/L peptone, 1 g/L acid hydrolysate of casein, 4 g/L agarose) containing 100 μL of the protoplast/PEG mixture evenly across 0.6% RG II plates containing 100 μg/mL hygromycin B.

### 2.6. Fluorescence Microscopy

Putative GFP- and DsRed-expressing endophyte transformants were examined using a Leica MZ FLIII fluorescence microscope (Leica Microsystems, Wetzlar, Germany) fitted with filters GFP2 and GFP3 with excitation filters at 480/40 and 470/40 nm, respectively, and barrier filters at 510 nm LP (Long Pass) and 525/50 nm, DsRed filter 546/10 nm and emission filter 565 nm LP.

Transformant colonies were examined for GFP and DsRed expression under a confocal microscope. Hyphae suspended in a drop of water were observed using an Olympus FluoView FV10i confocal microscope (Olympus, Tokyo, Japan). Filters (green-narrow with excitation maximum 473 nm, emission maximum 490–540 nm and TRITC with excitation maximum 552 nm and emission maximum 578 nm) were used to monitor GFP and DsRed expression.

Samples of endophyte infected plants were observed using a Leica M205 FA fluorescence stereo microscope (Leica Microsystems, Wetzlar, Germany) fitted with filters GFP2 and GFP3 with same excitation and barrier filters as above.

### 2.7. Seedling Inoculation of Transgenic Endophytes

Endophyte-devoid (E^−^) perennial ryegrass cultivar Alto seeds were obtained from Barenbrug Agriseeds, Christchurch, New Zealand. PCR-verified, fluorescent protein-expressing representatives from each reporter gene-containing endophyte strain (NEA12-GFP1, NEA12-DsRed9, E1-GFP2, and E1-DsRed4) were used for inoculations. A total of 60–75 seedlings were inoculated with each individual transgenic endophyte strain and 210–230 seedlings were co-inoculated with NEA12-GFP1 + NEA12-DsRed9, E1-GFP2 + E1-DsRed4, and E1-GFP2 + NEA12-DsRed9. Maintenance of inoculated plants were performed as previously described in [35]. Inoculated seedlings were incubated in a growth room (23 °C, 80 µMm^−2^ s^−1^, 8–16 h photoperiod) for 7 days and then transferred to the glasshouse and planted in 42-cell plant trays filled with potting mix.

### 2.8. Screening for the Presence of Endophytes in Planta

Three tiller samples (<0.5 cm) were harvested from the base of 6-month-old soil grown plants, followed by freeze-drying (Virtis Genesis, 25XL, Gardiner, NY, USA) for 48 h. DNA was extracted using the Qiagen MagAttract DNA Extraction kit (Qiagen, Hilden, Germany) as per manufacturer’s instructions. Presence of the reporter genes were assessed by real time amplification (2x Roche FastStart SYBR Green Master Mix (Roche Ltd., Mannheim, Germany), 200 nM primers (*gfp*-F: 5′-GGTGAACTTCAAGATCCGCC-3′, *gfp*-R: 5′-GAGGGTGTTCTGCTGGTAGT-3′: *DsRed*-F: 5′-GCGTGATGAACTTCGAGGAC-3′, *DsRed*-R: 5′-TTCACGCCGATGAACTTCAC-3′), 2 µL DNA template (5 ng/µL), in a total volume of 10 µL) using the BIORAD CFX96 Touch™ Real-Time PCR detection system (Bio Rad Laboratories Inc., Hercules, CA, USA) (95 °C 10 min, (95 °C 30 s, 60 °C 1 min) × 40, melt curve 60–95 °C (0.5 °C increments) for 5 min). Three technical replications for each sample were included. Plasmid DNA carrying *gfp* or *DsRed* reporter genes (0.5 ng) acted as the positive controls. Data were analysed by Bio-Rad CFX Manager software v.3.0 (Bio Rad Laboratories Inc., Hercules, CA, USA) using automatic threshold calling.

### 2.9. DNA Sequencing and Analysis of Transgene Integration Sites

#### 2.9.1. Illumina Sequencing

Genomic DNA was quantified using a Qubit Fluorometer (Thermo Fisher Scientific, Walthman, MA, USA) and library of E1-GFP2 was prepared using the Nextera™ DNA sample prep kit (Illumina, Inc. San Diego, CA, USA) in accordance with the manufacturer’s protocol. The library was quantified using the KAPA library quantification kit (KAPA Biosystems, Wilmington, MA, USA), and the insert size was measured with an Agilent 2100 bioanalyser (Agilent Technologies, San Diego, CA, USA). Sequencing was performed using Illumina MiSeq technology (Illumina, Inc. San Diego, CA, USA) according to the manufacturer’s protocol.

#### 2.9.2. MinION Nanopore Library Preparation and Sequencing

Genomic DNA was extracted, libraries were prepared, and sequencing performed as described in [6]. DNA was extracted from snap frozen endophyte mycelia using a cetyltrimethylammonium bromide (CTAB)-based extraction method. Genomic libraries were prepared and sequenced using ligation sequence preparation kit (SQK-LSK109; Oxford Nanopore, Oxford, UK).

#### 2.9.3. Processing of Sequencing Reads

All Miseq reads were filtered using a custom Python script. All MinIon reads were filtered and assembled as described in [6]. Sequence correction, trimming, assembly, polishing the scaffolded assembly using short-reads generated from Illumina Miseq sequencing platform was also performed as described in [6]. In brief, sequence correction, trimming, and assembly was performed using the long-read assembler Canu v.1.8 [38]. Scaffolded assembly was polished with genome assembly improvement and variant detection tool Pilon v. 1.23 [39] using Miseq reads.

Presence, copy number, length, start and stop coordinates, as well as orientation of *gfp*, *hph*, *gpd*P, *trpC*P, and *trpC*T in the endophyte genome were initially identified through nucleotide BLAST (Basic Local Alignment Search Tool) (version 2.2.25) analysis [40], which led to the identification of the pattern of T-DNA integration. Subsequently, junction sequences were identified and nucleotide BLAST against the transformation vector was performed to study their origin. The DNA sequences immediately upstream and downstream of the integration were analysed to determine associated microhomology and the genomic position of T-DNA integration in the endophyte genome.

## 3. Results

### 3.1. Construction of Binary Reporter Gene Vectors

The Gateway™-enabled destination vector (pEND0002) containing *hph* selectable marker cassette was used to permit insertion of reporter genes between constitutive control elements. The expression-specific construct plasmids (pEND-*DsRed*, pEND-*egfp*, and pEND-*sgfp*) were constructed by replacement of the Gateway RFA-A cassette in vector pEND0002 by the reporter genes *DsRed*, *egfp*, and *sgfp*.

### 3.2. A. tumefaciens-Mediated Transformation of Endophyte Mycelia

All binary vectors generated (pEND-*DsRed*, pEND-*egfp*, and pEND-*sgfp*) were transformed into *A. tumefaciens* cells of strains AGL1 and LBA 4404, and PCR analysis was used to confirm the presence of the transformed plasmids. Hygromycin B at 200 μg/mL was used for selection of putative transformants. Following co-cultivation with finely cut mycelium of NEA12 and E1, hygromycin B-resistant colonies developed 2–4 weeks after transformation. No growth was observed in the non-transgenic endophyte controls at this concentration of hygromycin.

Both *A. tumefaciens* strains produced hygromycin B-resistant transformants, but the number produced by AGL1 was five-times higher than for LBA 4404. Fifty-six transformants were obtained for NEA12. The number of individual putative E1 transformants was too large to be counted, due to high-density growth. Stable transformation events were confirmed by the ability of these putative transformants to grow effectively at high concentrations of hygromycin B (300 μg/mL).

### 3.3. PCR Analysis and Mitotic Stability Assessment of Reporter Gene-Containing Endophytes

A sub-set of randomly selected hygromycin B-resistant transformants from each endophyte strain (six SGFP, six EGFP, and six DsRed transformants) were analysed by PCR using primers specific for the *gfp*, *DsRed,* and *hph* genes. GFP and DsRed transformants, which produced the expected product sizes for *hph* (414 bp), *gfp* (440 bp), and *DsRed* (385 bp) genes, were identified.

*A. tumefaciens*-mediated transformation of coenocytic mycelia may result in mixtures of genetically distinct nuclei, both lacking and containing the integrated reporter gene. To minimise potential problems associated with chimeric expression of fluorescent proteins, protoplasts of PCR-positive reporter gene-containing endophytes were prepared and regenerated. One variant of GFP, SGFP, for each endophyte strain was used for further analysis. A total of 48 hygromycin B-resistant reporter gene-containing endophytes (12 from each of the reporter gene-containing endophyte strains NEA12:SGFP, NEA12:DsRed, E1:SGFP, and E1:DsRed) were analysed by PCR using primers specific to the *gfp*, *DsRed,* and *hph* genes. The expected PCR product sizes were observed for all transformants (Figure S1).

Hygromycin B-resistant transformants expressing either the GFP or DsRed fluorescent proteins were sub-cultured for a minimum of five cycles over a period of six months. All tested transformants maintained the ability to grow on selection. After 12 months of consecutive sub-culture, reporter gene-containing endophytes for both target strains continued to express the respective fluorescent proteins.

### 3.4. Visualisation of Reporter Gene Expression

**Figure 1:**
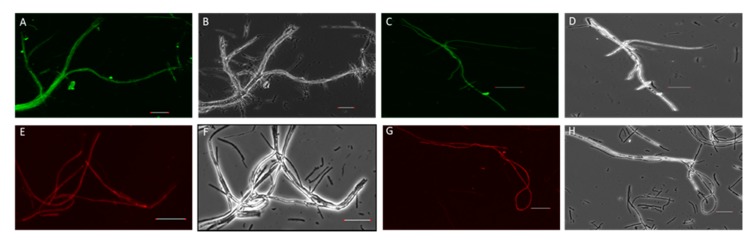
Confocal microscopy images of transgenic reporter mycelia (NEA12 and E1) expressing *sgfp* and *DsRed* genes. (**A**) NEA12 mycelia expressing SGFP. (**C**) E1 mycelia expressing SGFP. (**E**) NEA12 mycelia expressing DsRed. (**G**) E1 mycelia expressing DsRed. (**B**), (**D**), (**F**), and (**H**) are bright field images of (**A**), (**C**), (**E**), and (**G**), respectively. Scale bar = 40 µm.

DsRed- and SGFP-specific fluorescence were observed for all transformants (Figure 1). Young, actively growing hyphae showed strong expression of GFP or DsRed, while longer-established mycelium exhibited reduced or negligible expression.

### 3.5. In Planta Detection of Endophytes

Representatives of each reporter gene-containing endophyte strain were assessed for the ability to efficiently inoculate perennial ryegrass seedlings using an RT-PCR assay that targets *gfp* and *DsRed*. Initially, melt curve analysis was performed to confirm the specificity of primer annealing. Single sharp peaks were obtained, confirming primer specificity. Positive associations between ryegrass seedlings and reporter gene-containing endophytes were identified for NEA12-GFP1, NEA12-DsRed9, E1-GFP2, and E1-DsRed4. Infection frequencies obtained six months post inoculation are summarised in Table 2.

**Figure 2:**
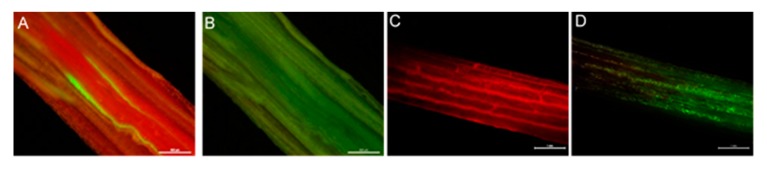
Fluorescence images of plant tissues inoculated with endophyte strain E1. (**A**) Expression of GFP at the base of a tiller. (**C**) Expression of DsRed in intercellular spaces of leaf sheath. (**B**,**D**) Corresponding bright-field images. Scale bar = 500 µM.

Endophyte-infected plants that tested positive for the *gfp* and *DsRed* transgene using RT-PCR analysis were further analysed for fluorescence in planta. GFP- and DsRed-specific fluorescences were detected, more specifically, in the basal meristem and intercellular spaces of leaf sheaths of plants infected with reporter gene-containing endophytes (Figure 2).

Secondly, to study the colonisation ability of endophytes of the same taxon or different taxa simultaneously in the same host, inoculation of complementary endophytes expressing GFP and DsRed was performed. Co-existence of GFP and DsRed expressing endophyte strains of the same taxon was identified, however GFP and DsRed expressing endophyte strains from different taxa were not observed to co-exist six months post inoculation (Table 3). 

### 3.6. DNA Sequencing and Analysis of Transgene Integration Sites

Endophyte strain E1 was chosen for further studies due to the beneficial properties of this endophyte strain, such as a relatively fast growth rate and high inoculation ability compared to other asexual *Epichloë* strains. One representative of E1-GFP was sequenced using Illumina MiSeq and Oxford Nanopore MinION sequencing for detailed analysis of T-DNA integration in the endophyte genome. The Illumina MiSeq run generated 2,033,256 reads and the Oxford Nanopore MinION run produced 466,415 reads for a total of 5.3 Gbp of data.

**Figure 3:**
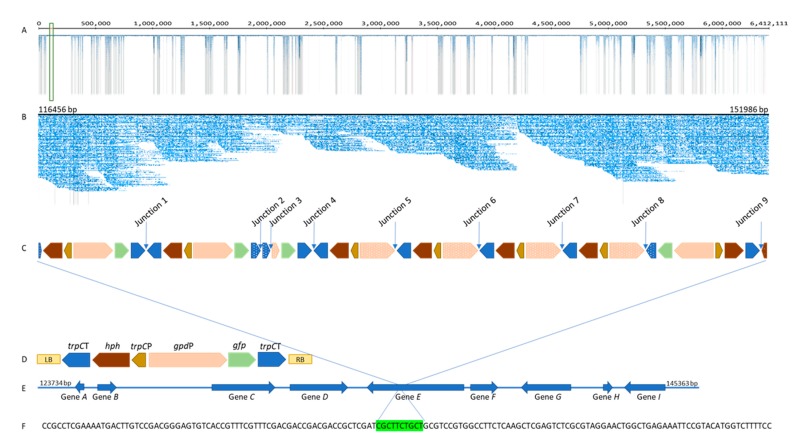
(**A**) Gydle output showing MinIon reads mapped to contig 3 (6,412,111 bp) of E1-GFP (integration site is highlighted in green box). (**B**) Gydle output showing MinIon reads mapped to the integration site. (**C**) Schematic representation showing the pattern of T-DNA integration in the E1 genome. Arrows indicate the junctions between T-DNAs and dotted blocks indicate truncations of more than 15 bp. (**D**) Schematic representation of the T-DNA region of the transformation vector, pEND-*sgfp,* showing relative position of the selectable marker (*hph*), promoter sequences (*gpd*P, *trpC*P), reporter gene (*gfp*), terminator sequences (*trpC*T), left border (LB), and right border (RB). (**E**) Schematic representation of the genes upstream and downstream of the integration site. (**F**) The 10 bp deletion in the E1 genome associated with the T-DNA event (deletion is highlighted in green).

BLASTN analysis, as described above, was used to identify the presence of the transgenes and their corresponding promoters and terminators, and consequently the T-DNA integration site in the E1 genome. Integration of multiple T-DNA into a single locus close to the telomere in the 6,412,111 bp contig 3 of E1-GFP was detected (Figure 3A), resulting in a 35,530 bp insertion from 116,456 bp to 151,986 bp (Figure 3B).

Co-integration of multiple T-DNA copies (10 T-DNA insertions) including four reporter gene cassettes and eight selectable marker cassettes with some degradations and rearrangements were identified (Figure 3C). With one exception, all T-DNA copies were organised in the same orientation as the T-DNA region of the transformation vector (Figure 3D). Full-length as well as truncated promoter, gene, and terminator sequences were observed. Interestingly, all *gfp* and *trpC*P insertions were found to be intact. One copy of *hph,* at the terminus of a T-DNA insertion, was truncated, while the other seven *hph* genes were intact. The presence of both intact and truncated copies of *gpd*P and *trpC*T may be due to the fact that most of T-DNA-T-DNA junctions were between *gpd*P and *trpC*T.

Three T-DNA copies (before junction 1, between junctions 1–2 and 8–9) contained *hph* and *gfp* gene cassettes including their corresponding promoters and terminators without internal T-DNA sequence deletions. However, their terminators were truncated, especially the first terminator at the start of integration. Breaks in the middle of T-DNA copies were identified for all other T-DNA copies, indicating some form of partial deletion during T-DNA integration.

T-DNA integration in the recipient E1 genome did not occur at the LB and RB sequences. For example, integration of one terminus occurred at *trpC*T and the other end at the *hph* gene. Short stretches of both *trpC*T and *hph* components were identified at these termini of integration; 10 bp compared to 775 bp long intact *trpC*T and 226 bp compared to intact 1020 bp long *hph*. Comparison between T-DNA end sequences and genome sequences at insertion sites revealed that T-DNA integration was associated with 3 bp microhomology between genomic DNA and one T-DNA terminus. Exact union between the T-DNA border and genomic DNA was observed at the other terminus.

Nine T-DNA-T-DNA junctions were observed at the single integration locus identified in the E1-GFP genome. The T-DNA-T-DNA junctions identified included two LB-RB junctions (1 and 4), four *trpC*T-truncated *gpd*P junctions (5, 6, 7, and 8), one between two truncated *trpC*T (2), one *gpd*P-truncated *trpC*T (3) junction, and one *trpC*T-truncated *hph* junction (9). No precise junction involving an intact left and right border was found. Furthermore, no vector backbone insertions or filler sequences were identified.

Junctions 1, 2, and 4 are between two *trpC*T. Analysis of these junctions identified that junction sequences consist of the three last bp of the LB to the start of *trpC*T of the *hph* cassette and first three bp of the RB, except junction 2 where the sequence between the LB and the start of *trpC*T of the *hph* cassette is missing. All three RBs identified (1, 2, and 4) displayed a breakpoint located three bases (TGA) into the border sequence. Between junctions 4 and 8, four replicates of the left part of the T-DNA region (*trpC*T-*hph*-*trpC*P-*gpd*P) were identified.

Junctions 5, 6, 7, and 8 were very similar and are between an intact *trpC*T and a *gpd*P with a 280 bp truncation, except at junction 2 where the *trpC*T terminator has a 239 bp truncation. These junction sequences were matched 100% to the vector sequence between *trpC*T and two (7 and 8) or three (5 and 6) bp before the start of the RB. The components of the *gfp* gene cassette, which are located after *gpd*P (*gfp* and *trpC*T), have been truncated at some stage of T-DNA integration and linked to *trpC*T of the *hph* gene cassette (the sequence from the LB to the start of *trpC*T is also truncated). Junctions 9 (between an intact *trpC*T and *hph* (226 bp)) and 3 (*trpC*T (320 bp) and *gpd*P (336 bp)) are unique.

T-DNA integration was identified at contig 3, which started with 17 copies of a telomeric repeat (TAACCC). The T-DNA has integrated into a gene-rich region of the E1 genome; analysis of T-DNA-genome junction sequences identified that T-DNA is integrated into a gene predicted to encode for a hydrolase (*Pochonia chlamydosporia* 170 hydrolase, α/β fold family protein (XM_018288623.1)) (Gene *E*) (Figure 3E). Further study of the genes immediately upstream and downstream of Gene *E* indicates that these genes are highly conserved, with closely related Clavicipitacae species such as *Metarhizium brunneum*, *M. acridumm* and *P. chlamydosporia* (Table S2). This insertion event is associated with a 10 bp deletion in Gene *E* of the E1 genome (Figure 3F).

## 4. Discussion

### 4.1. Vector Construction and A. tumefaciens-Mediated Transformation

GFP variants such as SGFP and EGFP have been shown to confer high levels of fluorescence in several fungal species, including *Cochliobolous heterostrophus, Ustilago maydis*, *F. oxysporum*, and *Phytophthora palmivora* [41,42,43,44]. Similarly, the codon-optimised version of DsRed has been expressed successfully in filamentous fungi such as *Verticillium albo-atrum*, *F. oxysporum*, *L. maculans*, *L. biglobosa*, *Oculimacula yallundae*, *O. acuformis*, *Penicillium paxilli*, and *Trichoderma harzianum* [13,45,46,47,48]. Constitutive and heritable expression of GFP fluorescence in transgenic organisms has provided an ideal system for assessment of organismal viability [49]. In addition, major advantages of the use of reporter genes to study the host-endophyte association include the ability to visually locate fungal mycelia in planta; hence, ideal for monitoring different stages of life cycle, provide fast real-time temporal resolution, facilitate rapid screening and discrimination of different inoculated strains, as well as to distinguish inoculated strains from naturally present plant-associated microorganisms [10,18,50]. Based on all these desirable qualities of fluorescent proteins, *sgfp*, *egfp*, and *DsRed* were selected as reporter genes for the present study.

Expression vectors containing the reporter genes were constructed using Gateway™ cloning and transformed using two *A. tumefaciens* strains, AGL1 and LBA 4404. Ability to induce tumours on particular plant species, which are the natural primary host, varies between *A. tumefaciens* strains, and it is unclear whether similar variation arises in respect to the capacity to also transform fungi [51]. In the present study, the transformation efficiency of the AGL1 and LBA 4404 strains differed substantially. AGL1 has previously been shown to be more efficient than other *A. tumefaciens* strains for transformation of both ascomycete and basidiomycete fungal species [52,53], consistent with the outcomes of this study. A further practical limitation of the use of LBA 4404 is a tendency to form aggregates in liquid culture, which makes accurate determination of optical density difficult [46].

The backbone vector used in this study was conveniently manipulated for multiple other purposes, in addition to the successful expression of GFP and DsRed. This included modification of the vector to express peramine in culture and in planta [35], as well as for construction of gene silencing vectors to identify the genes responsible for epoxy-janthitrem biosynthesis [6].

### 4.2. Molecular Analysis of Transformants

PCR-based analysis confirmed presence of *hph* and the corresponding reporter gene in transformants that were identified based on growth on antibiotic selection media following *A. tumefaciens*-mediated transformation.

### 4.3. Expression of Fluorescence Proteins in Culture

Visualisation of GFP and DsRed expression in reporter gene-containing endophytes showed that the level of fluorescence varied between individual transformants, potentially reflecting copy number and position effects. However, microscope-based examination revealed no gross differences in expression levels between the two GFP variants, *sgfp* (codon-optimised for higher plants [54]) or *egfp* (codon-optimised for mammals [54]). Growing hyphal tips always exhibited higher expression of GFP or DsRed presumably due to relatively high nuclear density. This observation may also be due to variable levels of transgene expression in some nuclei according to developmental stage or transgene silencing in non-dividing mycelial cells. In common with the present study, physical variation in the intensity of florescent protein expression has been observed for *Botrytis cinerea*, *Pyrenophora tritici-repentis,* and *Sclerotinia sclerotiorum* [42,55].

Expression of GFP and DsRed was stable in transformants of both endophyte strains and did not disappear after subsequent sub-culture over a period of twelve months. High rates of mitotic stability of *gfp* gene containing transformants of different fungal species generated by *A. tumefaciens-*mediated transformation have also been reported in other studies [56,57].

*A. tumefaciens*-mediated transformation of mycelia may result in cells that have genetically different nuclei, some containing and some lacking the integrated reporter gene. Therefore, protoplasts from PCR-positive transformants were prepared to mitigate the risk of analysing mycelia with chimeric nuclear content arising from both transformed and non-transformed nuclei. This strategy is vulnerable to the possibility that protoplasts may be multinucleate rather than uninucleate, which could inadvertently capture both transformed and non-transformed nuclei. However, sequence analysis confirmed the presence of single transformed nuclei in E1-GFP transformant. Purification of transformants has also been attempted by subculturing of hyphal tips and serial transfers on selective media [42]. However, homokaryotic mutants of *Monilinia fructicola* were not identified even after four rounds of single hyphal tip purification [58].

### 4.4. Inoculation Ability of Reporter Gene-Containing Endophyte Strains and in Planta Fluorescent Protein Expression

Fluorescent proteins provide a unique and visual phenotype for studying plant-microbe interactions without any external intervention, and so providing valuable information on in planta development of endophyte. The colonisation ability of a transgenic endophyte could be compromised by transgene insertion, especially if a critical gene is disrupted [59]. Therefore, one representative from each reporter gene-containing endophyte strain was assessed for their infection ability. Colonisation ability was observed for *gfp* and *DsRed* containing NEA12 and E1 endophyte strains six months after inoculation indicating vegetative stability of the host-endophyte association over time.

GFP- and DsRed- specific fluorescence of plants infected with transgenic endophytes was observed in basal meristems, as well as in intercellular spaces in which endophytes are abundantly located. Each individual plant infected with a transgenic endophyte strain showed normal growth in the glasshouse and stable expression of either GFP or DsRed was retained. The stability of the associations established provides evidence for the ultimate value of reporter gene-expressing endophytes for assessment of spatial and temporal changes during host colonisation and longer-term evaluation of symbiota through a time-course of plant growth and development to the next generation through the seeds, while emphasising the importance of access to endophyte strains of different taxa with different fluorescent protein expressions.

### 4.5. Co-Existence Ability of Reporter Gene-Containing Endophyte Strains

This study used endophyte strains that belong to same and different taxa which express GFP and DsRed to investigate co-existence potential of endophytes within the same host plant. Only a few studies have so far attempted to understand this phenomenon, and the mechanisms that govern an inability to co-exist within the same tiller are yet to be elucidated [60,61,62]. As part of the studies performed so far, different endophyte strains in the same host have been distinguished based on characteristics such as presence or absence of conidia, production of certain specific alkaloids, and RAPD (random amplification of polymorphic DNA) profiles [60,61]. However, none of these identification methods can provide real-time temporal resolution, as may be achieved by use of fluorescent proteins. No prior study of co-existence and distribution of multiple endophytes within an individual plant have used fluorescent-labelled endophytes, so the strains described here provide dynamic tools for a better understanding of the mechanisms involved during co-inoculation, co-existence, and competition between co-inoculated strains.

Co-inoculation events were identified for both NEA12-GFP1 + NEA12-DsRed9 and E1-GFP2 + E1-DsRed4 suggesting stable co-colonisation is possible within the same taxa. Only E1-GFP was identified for plants inoculated with both E1-GFP2 + NEA12-DsRed9. E1 has a faster growth rate and high inoculation ability compared to NEA12. These attributes may have enabled E1 to outcompete and become dominant. This characteristic of E1 has also been observed in other co-inoculation studies using different perennial ryegrass endophyte strains. This co-inoculation study was performed mainly to understand the potential of using these reporter gene-containing endophyte strains to study host-endophyte interactions. However, it would be beneficial to co-inoculate and analyse a greater number of plants to understand the co-infection ability of endophytes precisely. It has been found that hyphae of the dominant strain colonised primordial tillers arising from a dually infected plants, and co-existence disappeared over time, giving rise to plants in which all tillers contained a single strain [60].

### 4.6. Bioinformatic Analysis of Transgene Integration Sites

T-DNA integration is a complex process and its mechanism is not yet completely understood [32,63]. Understanding the complex nature of T-DNA integrations may help to develop and optimise transformation methods and conditions to achieve transformants with only intended modifications. Integration patterns of T-DNA in a small number of plant genomes have been studied in detail [31,64]. In the case of fungi, even though *A. tumefaciens*-mediated transformation has been used extensively for generation of transgenic strains, integration patterns of T-DNA in fungal genomes has not been characterised to the same extent [65,66,67]. A detailed understanding of T-DNA integration is essential to develop transformation methods to obtain a more desirable outcome, which is very important for both commercial and research purposes [25,32]. Traditionally, molecular techniques such as Southern blotting, PCR, and genome walking have been used for characterisation of T-DNA integration [21,57,68]. However, these approaches are not capable of identifying complex integration events [69]. Next-generation sequencing has been used more effectively for the analysis of T-DNA integration patterns. Although there are limitations such as difficulties in assembling large repeat structures and other complex regions accurately [64]. Further, due to the short reads generated, these sequencing methods only identify flanking sequences of T-DNA and genomic DNA [64]. More recently, the combination of next-generation sequencing and third-generation techniques such as PacBio and MinIon sequencing have been used to analyse T-DNA integration events and associated genomic variations in the recipient genome with higher precision and in greater detail [31,64]. However, to date, studies using long read sequencing to analyse T-DNA integration events are limited to a few plant species such as *Arabidopsis* and *Betula* (birch) [31,64].

This study used a combination of second- and third-generation sequencing (Miseq and MinIon, respectively) to identify and characterise T-DNA integration in the endophyte strain, E1-GFP. Insertion of 35 kb containing 10 T-DNA copies was identified in contig 3 of E1-GFP, which is homologous to chromosome III of *E. festucae* strain Fl1, 6 Mb [70,71]. For some fungi, the number of T-DNA copies integrated may depend on the *A. tumefaciens*-based transformation method. In *A. aculeatus*, *Blastomyces dermatitidis*, and *Suillus bovinus*, a high concentration of *A. tumefaciens* cells resulted in multiple copy integrations, while low concentrations resulted predominantly in single copy integrations [72]. However, *A. tumefaciens* concentration exerted no obvious influence on T-DNA copy number in *F. oxysporum* [73]. Occurrence of transformants with single or multiple copy T-DNA integration patterns has also been shown to depend on the addition of acetosyringone to the *A. tumefaciens* pre-culture and the length of the co-cultivation period [22,74]. Presence of multiple T-DNA inserts in the E1-GFP strain analysed could potentially be due to the nature of the transformation conditions used in this study. These conditions included a co-cultivation time of 72 h and pre-treatment of the *A. tumefaciens* cells with acetosyringone prior to co-cultivation with endophyte mycelia. T-DNA may have been sequentially integrated into a single recipient genome through more than one round of transformation.

T-DNA truncations at both the LB and RB as well as deletions of borders were observed in the present study. Three right-border breakpoints (with only the first 3 bp of RB) were detected in three of the nine junctions while two left-border breakpoints (with only the last 3 bp) were detected in two of nine junctions identified. Consistent with this observation, the most commonly identified right-border breakpoint is at the first three nucleotides (TGA) of the RB sequence [24]. This is consistent with the expected nick point between the third and fourth nucleotide of the 25 bp repeat [24]. Truncations of T-DNA may arise either due to recognition of non-border as border sequences, with subsequent nicking of the T-DNA at these locations, or to exonuclease digestion of the T-DNA ends prior to integration or breakage of the T-DNA during transfer [24]. In *M. oryzae*, the LB sequence is truncated at a high frequency when compared to the RB [20,75]. In contrast, conservation of both borders has been observed for transformants of *L. maculans* [67].

Four *gfp* cassettes were present in ten T-DNA copies identified. Three of them contained intact *gfp* genes and intact *gpd*P promoters. Only one of those three cassettes have full length *trpC*T while the other two have truncated *trpC*T (<250 bp truncations). The fourth *gfp* gene cassette has a full-length *gfp* gene with very short fragments of corresponding promoter and terminator and therefore may not be transcribed. The other six T-DNA copies contained *hph* cassettes with truncated *gpd*P. Deletions of T-DNA were observed not only at the ends, but also in the middle of the T-DNA. Interestingly, four replicates of the left part of the T-DNA (*trpC*T-*hph*-*trpC*P-*gpd*P) were observed between junctions 4 and 8 proving the complex nature of T-DNA integration. This could be due to occasional random nicking of the T-DNA and nuclease digestion of the T-DNA ends [24]. The integration of DNA with part of the T-DNA fragment missing or with rearrangements has been identified frequently in plant genomes [64]. This complex pattern of T-DNA integration was further confirmed by long read sequences that span the entire 35 kb integration.

Integration of T-DNA requires a DNA repair pathway. In fungi, the main pathway is non-homologous end-joining (NHEJ), however targeted integration of the T-DNA by homologous recombination (HR) is also possible in species such as yeast [76,77]. NHEJ requires a small microhomology of up to 5 bp between T-DNA and host genomic DNA whereas homologous recombination requires extensive DNA sequence homology [76,77,78]. During NHEJ, some rearrangements such as deletions of a few nucleotides and/or duplications have been observed at double strand break sites in plants [77]. In this study, microhomology of 3 bp was observed at the junction between one T-DNA terminus and endophyte genome, suggesting that T-DNA integration in *Epichloë* does not depend upon long stretches of homology at cross-over points, similarly to that observed for *M. oryzae* [75]. No microhomology of T-DNA end and genome was observed at the other end of the integration. Microhomology between T-DNA ends and the genomic DNA has also been reported for other fungi such as *S. cerevisiae* and *C. neoformans* [20,27]. Identification of a 10 bp deletion of endophyte genome sequence around the insertion site in this study is consistent with observations for other fungi including *M. oryzae* and *C. neoformans*. Similar to the results of the current study, most of the identified deletions were less than 20 bp in length in these fungi [20,75].

Analysis of genomic flanking sequences in the present study identified that the T-DNA has integrated in a gene-rich region of E1 genome and more specifically into a gene predicted to coding for a hydrolase. Still, it is debatable whether T-DNA integration targets certain genomic regions or it happens in a completely random manner [79]. Greater susceptibility of regions upstream from genes, as well as intergenic regions, to T-DNA integrations has been reported in the pathogenic fungus *H. capsulatum* [21]. In contrast, relatively even distribution of T-DNA integration events throughout the genome was observed for *S. cerevisiae*, *L. maculans,* and *M. grisea* [28].

This study revealed the complex nature of T-DNA integrations in fungi through analysis of a single transformant. Multiple T-DNA insertions with truncations of different lengths were identified with precise detail using a combination of long read and short read sequence data. These outcomes show the need for more thorough study of T-DNA integration in fungal genomes to better understand the process of T-DNA integration. This study has implications for genome modification, including genome editing, of fungal genomes using *A. tumefaciens*-mediated transformation and can further be explored to understand other structural variations of fungal genomes, which can be caused by T-DNA insertions.

## 5. Conclusions

A new set of fluorescent protein expression vectors were generated to study biological processes in endophytic filamentous fungi. Derivatives of endophyte strains NEA12 and E1 that express GFP and DsRed were generated and characterised in detail using a range of analytical techniques. Stable integration and expression of transgenes in endophytic fungi provides an efficient tool for exploration of the ability of endophytes to colonise plant tissue, to establish new inoculation methods and for the study of numerous aspects of host-endophyte and endophyte-endophyte interactions. A more detailed understanding of the mechanisms controlling endophyte co-existence will permit establishment of artificial symbioses between endophytes and host grasses that are capable of providing a larger range of benefits to host plants, through combinations of beneficial properties such as production of complementary bioprotective alkaloids for pest and disease control. Sequence analysis of T-DNA integration emphasizes the importance of development and optimisation of transformation protocols as well as screening the transformants in detail, most importantly in technologies such as foreign-DNA free genome editing. This is the first study to use a combination of second- and third-generation DNA sequencing methods to analyse copy number, sites, and exact structures of T-DNA integration events in fungal genomes with precise detail.

## Figures and Tables

**Table 1:** Properties of selected asexual *Epichloë* endophytes used for transgenic modification.

Endophyte Strain	NEA12	E1
Taxon ^a^	*Lp*TG-3	*Lp*TG-4
Origin ^b^	*Lolium perenne* (France)	*F. rubra* ssp. *commutate* (unknown)
Ploidy Level ^c^	haploid	haploid
Mating Type Idiomorph ^d^	*MTB*	*MTA*
Number of Mating Type Genes ^e^	1	3
Alkaloid Biosynthesis Profile ^f^	epoxy-janthitrems	epoxy-janthitrems
Growth Rate ^g^	Slow	Fast

^a,b,c,d,e,f^ [8]. ^g^ Compared by mycelial growth on PDA in 22 °C in the dark.

**Table 2:** Infection frequencies obtained for different reporter gene-containing endophyte strains.

Endophyte Strain	NEA12-GFP1	E1-GFP2	NEA12-DsRed9	E1-DsRed4
Number of plants analysed	60	60	49	57
Infection frequency (%)	6.3	14.5	1.6	11.6

**Table 3:** Infection frequencies obtained for co-inoculated endophyte strains.

Endophyte Strains	Number of Plants Analysed	Infection Frequency (%)		
		Single Reporter Gene (*gfp*)	Single Reporter Gene (*DsRed*)	Double Reporter Genes (*gfp + DsRed*)
NEA12-GFP1 + NEA12-DsRed9	202	4.5	1	0.9
E1-GFP2 + E1-DsRed4	217	7.8	18.9	2.8
E1-GFP2 + NEA12-DsRed9	213	16.1	0	0

## Supplementary Materials

The following are available online at https://www.mdpi.com/2076-2607/8/1/54/s1.
