# Supplementary material for: Generation of Epichloë Strains Expressing Fluorescent Proteins Suitable for Studying Host-Endophyte Interactions and Characterisation of a T-DNA Integration Event
Source: Microorganisms. 2019 Dec 27;8(1):54. doi: 10.3390/microorganisms8010054 (PMC7023320; doi:10.3390/microorganisms8010054)
Supplement: Supplementary file 1 [file microorganisms-08-00054-s001.zip › Supplementary files/Hettiarachchigeetal.-EndoReporterTransgenePap-Microorganisms-Figure S1.docx]

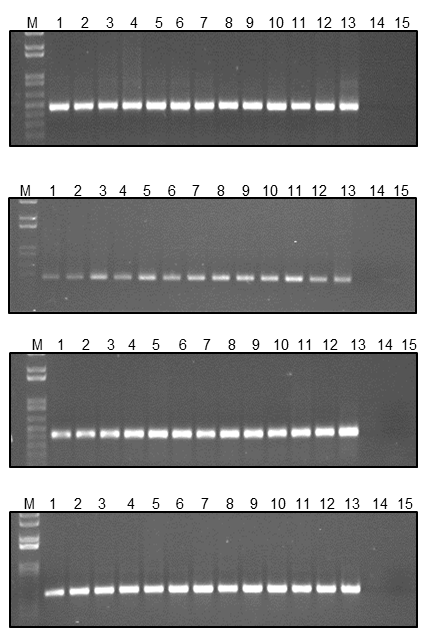

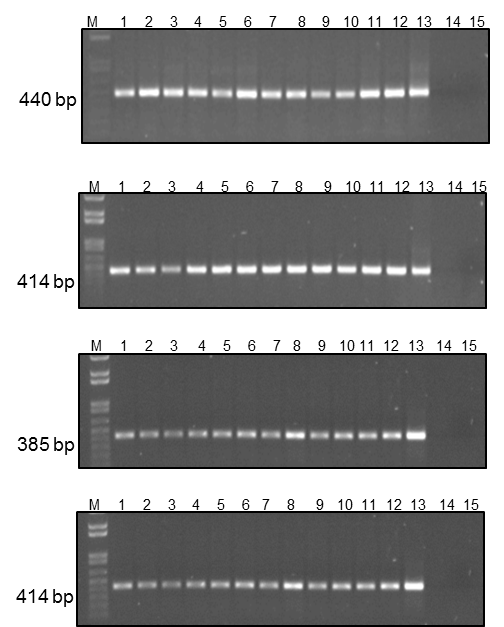


E1

NEA12

*hph*

*hph*

*DsRed*

A

B

C

D

E

F

G

H

*gfp*

**Figure S1**

PCR analysis of putative reporter gene-containing endophytes carrying *gfp* and *DsRed* genes. (A) and (E) Primers specific for *gfp*. Lane M: 1kb plus marker (Invitrogen). Lane 1-12: Transgenic endophytes expressing GFP. Lane 13: Positive control pEND-*sgfp*. Lane 14: Negative control (H_2_0 control). Lane 15: Non-transgenic endophyte. (B) and (F) Primers specific for *hph*. Lane M: 1kb plus marker (Invitrogen). Lane 1-12: Transgenic endophytes expressing GFP. Lane 13: Positive control pEND0002. Lane 14: Negative control (H_2_0 control). Lane 15: Non-transgenic endophyte. (C) and (G) Primers specific for *DsRed*. Lane M: 1kb plus marker (Invitrogen). Lane 1-12: Transgenic endophytes expressing DsRed. Lane 13: Positive control pEND-*DsRed*. Lane 14: Negative control (H_2_0 control). Lane 15: Non-transgenic endophyte. (D) and (H) Primers specific for *hph*. Lane M: 1kb plus marker (Invitrogen). Lane 1-12: Transgenic endophytes expressing DsRed. Lane 13: Positive control pEND0002. Lane 14: Negative control (H_2_0 control). Lane 15: Non-transgenic endophyte.
