# Supplementary material for: Generation of Epichloë Strains Expressing Fluorescent Proteins Suitable for Studying Host-Endophyte Interactions and Characterisation of a T-DNA Integration Event
Source: Microorganisms. 2019 Dec 27;8(1):54. doi: 10.3390/microorganisms8010054 (PMC7023320; doi:10.3390/microorganisms8010054)
Supplement: Supplementary file 1 [file microorganisms-08-00054-s001.zip › Supplementary files/Hettiarachchigeetal.-EndoReporterTransgenePap-Microorganisms-Table S2.docx]

**Table S2**

Accession numbers for closest matches in the Genbank database for genes predicted for E1 near the T-DNA integration site.

| Genes predicted for E1 | Accession numbers | | |
| --- | --- | --- | --- |
|  | *M. brunneum* | *M. acridum* | *P. chlamydosporia* |
| *A* | XM_014685036.1 | XM_007813894.1 | XM_018288642.1 |
| *B* | XM_014685035.1 | XM_007813893.1 | XM_018288643.1 |
| *C* | XM_014684557.1 | - | XM_018288589.1 |
| *D* | XM_014684078.1 | XM_007813848.1 | XM_018288620.1 |
| *E* | XM_014684080.1 | XM_007813850.1 | XM_018288623.1 |
| *F* | XM_014684081.1 | XM_007813851.1 | XM_018288624.1 |
| *G* | XM_014684082.1 | XM_007813852.1 | XM_018288625.1 |
| *H* | - | XM_007813853.1 | XM_018294647.1 |
| *I* | XM_014684084.1 | XM_007813854.1 | XM_018288626.1 |
