# Supplementary material for: Generation of Epichloë Strains Expressing Fluorescent Proteins Suitable for Studying Host-Endophyte Interactions and Characterisation of a T-DNA Integration Event
Source: Microorganisms. 2019 Dec 27;8(1):54. doi: 10.3390/microorganisms8010054 (PMC7023320; doi:10.3390/microorganisms8010054)
Supplement: Supplementary file 1 [file microorganisms-08-00054-s001.zip › Supplementary files/Hettiarachchigeetal.-EndoReporterTransgenePap-Microorganisms-Table S1.docx]

**Table S1.** Plasmids used and constructed in this study.

| Plasmid | Origin/Reference | Characteristics |
| --- | --- | --- |
| pPZP200 | [1] | Binary expression backbone |
| pEND0001 | This study | pPZP200 containing *hph* cassette |
| pEND0002 | This study | Destination vector |
| pDONR^TM^221 | Invitrogen^TM^ Life Technologies | Donor vector |
| pEND0003 | This study | Entry clone |
| pEND0004 | This study | Entry clone |
| pEND0005 | This study | Entry clone |
| pEND-*DsRed* | This study | Expression clone containing *DsRed* |
| pEND-*egfp* | This study | Expression clone containing *egfp* |
| pEND-*sgfp* | This study | Expression clone containing *sgfp* |

1. Hajdukiewicz, P.; Svab, Z.; Maliga, P., The small, versatile pPZP family of *Agrobacterium* binary vectors for plant transformation. *Plant Molecular Biology* **1994,** *25* (6), 989-994.
